# The Impact of Environmental Regulations on Pollution and Carbon Reduction in the Yellow River Basin, China

**DOI:** 10.3390/ijerph20031709

**Published:** 2023-01-17

**Authors:** Chengqing Liu, Dan Yang, Jun Sun, Yu Cheng

**Affiliations:** 1 School of Economics, Shandong Normal University, Jinan 250358, China; 2 Institute for Carbon Neutrality, Shandong Normal University, Jinan 250358, China; 3 College of Geography and Environment, Shandong Normal University, Jinan 250358, China

**Keywords:** Yellow River Basin, environmental regulation, pollution reduction and carbon reduction, inverted “U”

## Abstract

Based on data from 69 cities in the Yellow River Basin from 2006–2018, this paper examines the impact of environmental regulations on the integrated management of air pollution and CO_2_ emissions and its mechanism of action using a two-way stationary model. The results found that: (1) The impact of environmental regulations on air pollution and CO_2_ emissions in the Yellow River Basin has an inverted U-shaped trend, the intensity of regulation is still on the left side of the inverted U-shaped curve, and the inflection point has not yet appeared. (2) Environmental regulations suppressed air pollution and CO_2_ emissions by adjusting industrial structure, promoting technological innovation, and improving energy efficiency, but the current intensity of regulation is not sufficient to make the three paths fully effective. (3) The pollution and carbon reduction effects of environmental regulations are more significant in areas with higher marketization and resource dependence, national urban agglomerations, and the middle reaches of the Yellow River Basin. However, environmental regulations in other regions only show significant pollution reduction effects, and there is still more room for improving carbon reduction governance. Therefore, the Yellow River Basin should strengthen environmental regulations to promote ecological governance and high-quality development.

## 1. Introduction

With the rapid growth of China’s economy, resource and environmental problems have become increasingly prominent in recent years. Despite various arrangements to improve environmental quality, China, as a major global polluter, still faces great domestic and international environmental pressures. As an important ecological barrier and economic belt in China, the Yellow River Basin’s environmental governance and green transformation are related to China’s economic and social development and ecological security (quoted from General Secretary Xi Jinping’s speech at the “Symposium on Ecological Protection and High-Quality Development of the Yellow River Basin” in September 2019). In the report of the 20th National Congress of the Communist Party of China, “promoting ecological protection and high-quality development in the Yellow River Basin” was regarded as an important part of regionally coordinated development. Later, the promulgation of the *Yellow River Protection Law of the People’s Republic of China* provided legal protection for it. The introduction of this law and a series of laws, regulations, and environmental protection programs from the central to local governments show that environmental governance in the Yellow River basin was imminent. In reality, the ratio of good days of air quality in the Yellow River basin in 2020 will be 7.4% lower than the national average, and the total carbon emissions in the basin will account for 1/3 of the national total. The situation of air pollution and carbon emission control is severe. At present, more than 50% of the resource-based and old industrial cities and 80% of the coal chemical enterprises in China are still distributed in the Yellow River basin (according to the “Yellow River Basin Ecological and Environmental Protection Plan” in June 2022). Problems such as the heavy energy structure, the low degree of resource development and utilization, the lagging development of new and high-tech and strategic emerging industries, and the ineffective conversion of kinetic energy from old to new make its environmental governance face many difficulties. In this context, it is particularly important for the government to formulate a reasonable environmental regulation strategy, which can provide a reference for further developing green transition paths. Then, can environmental regulation in the Yellow River basin achieve the goals of reducing pollution and carbon emissions? Through what channels? Are there differences due to different regional characteristics? Answering these questions has important theoretical and practical significance for the Yellow River Basin to formulate more efficient and reasonable environmental policies to promote green economic growth.

Most studies show that environmental regulations have significant effects on pollution reduction and carbon reduction, which significantly improve environmental quality [1,2]. Specifically, in the research on the effect of pollution reduction, Shapiro and Walker (2018) found that environmental regulation brought down air pollutant emissions from U.S. manufacturing by increasing implicit pollution taxes on firms [3]. Zhang et al. (2020) believed that the government’s administrative regulations on environmental protection suppressed the emissions of atmospheric pollutants [4]. Greenstone and Hanna (2014) assessed India’s environmental regulations with a difference-in-differences design and found that air pollution regulations were associated with substantial improvements in air quality [5]. Other relevant studies show that the taxation system is a relatively effective policy tool for pollution control in China [6,7]. In the research on carbon reduction effect, Cheng et al. (2016) used a computable general equilibrium model to predict the impact of the low-carbon policy in Guangdong Province, China, and predicted that CO_2_ emissions in Guangdong Province in 2020 would be reduced to two-thirds of those in 2010 [8]. Liao et al. (2015) took the carbon emissions trading policy in Shanghai as the research object, and the empirical results showed that Shanghai’s carbon emissions trading policy was conducive to regional carbon emission reduction [9]. Xuan et al. (2020) found that China’s carbon emissions trading policy can significantly reduce the intensity of CO_2_ emissions and promote carbon emission reductions [10].

Of course, some scholars hold the opposite opinion and support the view of the “green paradox” effect in terms of environmental regulations. They believe that the current environmental regulation policy has not achieved the goal of pollution reduction [11], and instead, it increased the pollution emissions of enterprises [12]. Krass et al. (2013) argue that environmental taxes can put pressure on production costs, squeeze innovation inputs, and lead to increased pollutant emissions [13]. Zhang and Wang (2022) found that China’s provincial energy conservation policies and comprehensive resource utilization policies did not significantly curb CO_2_ emissions [14]. In addition, some scholars believe that the relationship between environmental regulation and air pollutants or CO_2_ emissions is not a simple linear one, but presents different fluctuation characteristics, such as a U-shape (reduction-increase), inverted U-shape (increase-reduction), or N-shape [15,16,17], meaning that for environmental regulation to take effect, there is a threshold. As the intensity of environmental regulation increases, the effects switches between the “green paradox” effect and the “forced emission reduction” effect.

Most scholars now study the impact of environmental policies on air pollutants or CO_2_ emissions from a single perspective. There are also a few studies that investigated the impact of environmental policies on the collaborative governance of pollution reduction and carbon reduction. Du and Li (2020) found that China’s environmental regulations promoted synergistic reductions in corporate pollutant and greenhouse gas emissions [18]. Zhao et al. (2022) used the PSM-DID method to study the effect of the Sulfur dioxide Emissions Trading Pilot Scheme (SETPS) on the reduction of corporate carbon emissions, and found that SETPS had a dual effect of suppressing corporate carbon emissions and other pollutant emissions [19]. Zhang and Zhang (2020) studied the carbon emissions trading market in China’s power sector and found that carbon trading policies can reduce both CO_2_ emissions and PM2.5 emissions [20]. The results of Li et al. (2021) also suggest that China’s carbon market pilot (CMP) can achieve the policy goal of carbon emission reduction while also providing a more efficient means of controlling air pollution [21].

However, most of the research areas of such literature are at the city, industry, or enterprise level, and few studies have investigated the effects of environmental regulation on pollution and carbon reduction for different agglomeration patterns. In addition, the effects of environmental regulation are uncertain and have different effects in different periods or regions, so the relevant studies often lack practical significance. A river valley or basin is often an area with a concentration of cities, so it is necessary to study a river valley as a whole to better optimize and integrate the resources in such a region. With regard to the ecological environment protection and high-quality development of the Yellow River Basin, the current research mainly involves the temporal and spatial evolution of air pollution and carbon emissions [22,23], resource protection and ecological governance [24], green development efficiency and realization paths [25,26], etc. Few studies have investigated the impact of environmental regulations on air pollution reduction and carbon reduction in the Yellow River Basin.

Based on this understanding, this paper uses the two-way fixed effects model to conduct an empirical study on the data of 69 prefecture-level cities in the Yellow River Basin from 2006 to 2018, explore the effectiveness of their environmental regulations on air pollution and CO_2_ emissions, and conduct a comparative analysis on the effects of pollution and carbon reduction. At the same time, it further investigates the mechanism and heterogeneity of environmental regulations in the Yellow River Basin. This paper makes three possible contributions to the literature. First, different from the studies on a national or provincial scale in the existing literature, this paper discusses the environmental regulation of the Yellow River Basin on air pollution and CO_2_ emissions from a more detailed urban scale, further enriching the perspective of relevant studies. Second, the impact of environmental regulations in the Yellow River Basin on air pollution and CO_2_ emissions will be analyzed in a unified framework to more comprehensively explore the governance effect of environmental regulations, and thus provide a theoretical reference for the formulation of subsequent pollution prevention policies. Finally, according to the analysis results of the function path and heterogeneity of environmental regulations, this paper can provide a reference for the Yellow River Basin to find the right entry point for pollution and carbon reduction, and implement differentiated management according to local conditions.

The structure of the remainder of this paper is as follows: Section 2 is the overview of the study area; Section 3 discusses the model building and data; Section 4 contains the benchmark regression results and robustness test; Section 5 is the transmission mechanism identification and heterogeneity analysis of environmental regulation; and Section 6 discusses the conclusions and policy recommendations.

## 2. Overview of the Study Area

### 2.1. History of Environmental Protection

In 2006, China promulgated the “11th Five-Year Plan” for the development of the western region, which proposed the development concept of emphasizing both development and conservation and giving priority to conservation. It can be seen that the ecological issues were not mainly “protection” but “development” and “conservation” of ecological resources at that time, which led to the reality that most areas are focused on resource development at the expense of environmental protection. Subsequently, from the 12th Five-Year Plan for Western Development promulgated in 2012 to the 13th Five-Year Plan in 2017, the emphasis on ecological protection has been gradually strengthened. However, the bulletin on the State of China’s Ecosystem in 2018 shows that the overall ecological environment quality of key national ecological function areas deteriorated by 1.9 percent from 2016 to 2018. Further research revealed that on the issue of development and protection of environmental resources, the rate of destruction slightly exceeded the rate of protection.

In September 2019, the ecological protection and high-quality development of the Yellow River Basin was elevated to China’s national strategy, indicating a new phase of environmental management in the Yellow River Basin. During this period, China has actively addressed water pollution in the Yellow River Basin and promoted cross-regional cooperation in environmental management. Despite the continuous introduction of policies related to environmental protection in the Yellow River Basin, the relatively fragmented governance model, lagging social governance capacity, and fragile ecological environment in the region have severely constrained the high-quality development of the Yellow River Basin. For this reason, it is particularly important to evaluate the effectiveness of environmental governance in the Yellow River Basin so as to provide lessons for future environmental governance.

### 2.2. Study Area Delineation

**Figure 1:**
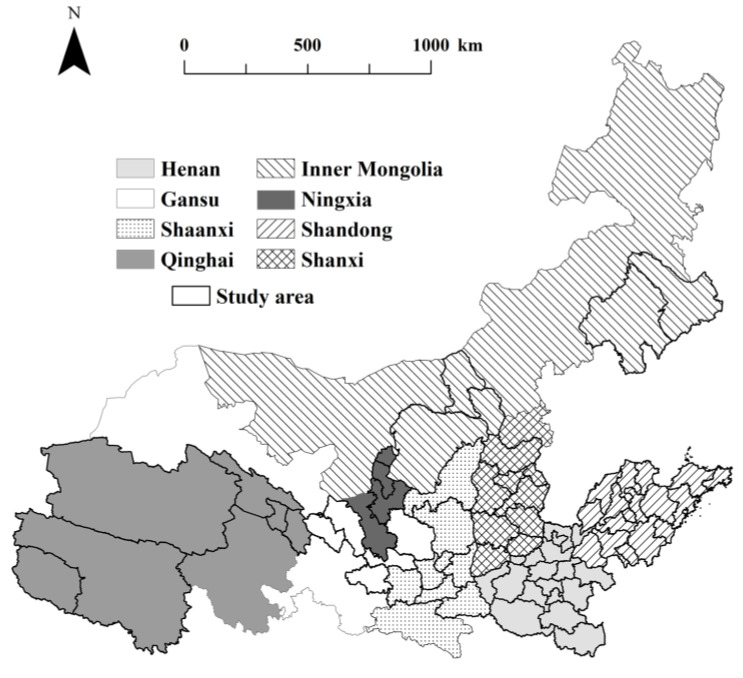
Regional location map of the Yellow River Basin.

The Yellow River is the second largest river in China and the fifth largest river in the world, with a total length of 5464 km and a basin area of 752,400 km^2^, spanning three major regions in China: the eastern, central, and western regions, which is an important economic zone. In 2019, the Yellow River Basin had a total population of 442 million, accounting for 31.58% of the country and the gross regional product was CNY 24.74 trillion, accounting for about 25% of the national GDP. According to the division method of the Yellow River Conservancy Commission and based on the natural demarcation points and considering that the implementation of environmental regulation policy is territorial, a total of 69 cities in eight provinces and regions of Qinghai, Gansu, Ningxia, Inner Mongolia, Shaanxi, Shanxi, Henan, and Shandong through which the Yellow River flows were selected as the study area, as shown in Figure 1.

## 3. Model Building and Data

### 3.1. Model Building

The two-way fixed effects model is a model with both “individual effects” and “time effects”. The data in this paper are balanced panel data, and for balanced short panel data, a fixed effects model is usually used. For the problem of this paper, since each city is different, there may be omitted variables that do not change over time, i.e., there is an “individual effect” and there may be a “time effect” that does not change with individual heterogeneity. Therefore, in this paper, a two-way fixed effects model was developed using Stata16.0. In addition, it was found that the impact of environmental regulations on air pollution and CO_2_ emissions may not be a simple linear relationship. Therefore, with reference to previous relevant studies, the model incorporates the primary and secondary items of environmental regulation. The model design is as follows:

(1) $$lnpoll_{it}=\partial_{0}+\partial_{1}ER_{it}+\partial_{2}ER_{it}{}^{2}+\partial_{3}lnpgdp_{it}+\partial_{4}lnfd_{it}+\;\partial_{5}lnopen_{it}+\partial_{6}lnpop_{it}+\partial_{7}gov_{it}+\varepsilon_{i}+\varepsilon_{t}+\varepsilon_{it}$$

(2) $$lnco_{2_{it}}=\beta_{0}+\beta_{1}ER_{it}+\beta_{2}ER_{it}{}^{2}+\beta_{3}lnpgdp_{it}+\beta_{4}lnfd_{it}+\;\beta_{5}lnopen_{it}+\beta_{6}lnpop_{it}+\beta_{7}gov_{it}+\varepsilon_{i}+\varepsilon_{t}+\varepsilon_{it}$$

where i and t represent the city and year, respectively; poll and $co_{2}$ represent air pollutants and carbon dioxide emissions, respectively; ER represents government environmental regulation; pgdp represents the economic development level; fd represents the financial development level; open represents the degree of opening to the outside world; pop represents the total population at the end of the year; gov represents government research and development (R&D) investment; $\varepsilon_{i}$ and $\varepsilon_{t}$ represent the fixed effects of city and year, respectively; and $\varepsilon_{it}$ represents the random error term. Individual missing data are supplemented by the interpolation method. At the same time, in order to mitigate the influence of heteroscedasticity, the data is in the form of natural logarithm except for the government R&D investment (gov). The estimated coefficients $\partial_{1}$, $\partial_{2}$, $\beta_{1}$, and $\beta_{2}$ reflect the pollution reduction effect and carbon reduction effect of environmental regulations, which are the estimation coefficients that are focused on in this paper. If the first order term coefficient of environmental regulation $\partial_{1}$ is positive, and the second order term coefficient $\partial_{2}$ is negative, and both are significant, it shows that the environmental regulation of the Yellow River Basin and air pollution emissions are in the inverted U-shaped relationship, otherwise, it is in the U-shaped relationship. $\beta_{1}$ and $\beta_{1}$ follow the same rule.

### 3.2. Description of Variables and Sources of Data

Since the data of industrial wastewater and sulfur dioxide emissions in some cities can be retrieved until 2018 and considering the availability and completeness of the data, this paper selected 2006–2018 as the research period.

Explained variable: Including air pollutant emission (poll) and CO_2_ emission data. Among them, since industrial SO_2_ accounts for a high proportion of urban air pollution in China [27], industrial SO_2_ was selected as the main air pollutant for analysis, and the data was retrieved from the China Urban Statistical Yearbook; CO_2_ emission data are from the China Carbon Emission Accounts and Datasets (CEADs).

Core explanatory variables: As for the measurement of environmental regulation indicators (ER), the amount of pollutant discharge fee [28], the amount of investment in environmental pollution control [29], or the comprehensive indicators for the conversion of multiple pollutant emissions are commonly used in the literature [18]. This paper used the comprehensive index of various pollutant emissions (industrial SO_2_, industrial wastewater, and industrial smoke and dust) to measure the intensity of environmental regulation [18]; as the three pollutants are non-additive and are measured in different units, the emissions of each pollutant per unit of economic output are dimensionless and were processed according to the entropy method, and the respective weight coefficients of each year were calculated, and then the environmental regulation intensity of each city was obtained by weighted summation. All other things being equal, the higher the emissions per unit of output, the more lenient the environmental regulations in that area. That is, emissions per unit of output are negatively correlated with the strength of environmental regulations. To facilitate the discussion of the results, the measurements were converted to their respective inverse in this paper.

Control variables: Combined with the existing research [30,31,32,33], this paper also added a group of control variables to the benchmark regression model to mitigate the bias of missing variables as much as possible. Specifically, it included the level of economic development (pgdp), expressed in actual per capita GDP. The larger the economic scale is, the more output will be generated, which will have a direct impact on air pollutant and CO_2_ emissions; the financial development level (fd) is expressed in the per capita loan limit of financial institutions. Financial development can provide financial support for enterprises, which may lead to energy consumption through enterprise scale expansion, or promote green transformation by encouraging green technology research and development and cleaner production. The opening degree (open) is expressed by the total import and export trade of the region in the year (according to the total import and export data (USD 10,000) in the Statistical Yearbook of each city; it is converted into CNY by multiplying by the average exchange rate of the year). The import and export trade between countries transfers the pollution and CO_2_ generated by production activities, which can also affect the emissions of these two kinds of pollutants by affecting the local industrial structure. The resident population at the end of the year is represented by (pop); population concentration may cause excessive production scale and rising congestion costs, and then accelerate energy consumption and promote pollutants and CO_2_ emissions. The R&D investment by the government is (gov), expressed by the proportion of government expenditure on science and education in financial expenditure. Through R&D investment, local governments can improve the efficiency of scientific and technological innovation of enterprises and achieve the goal of energy conservation and emission reduction. The data is from the China Urban Statistical Yearbook and the database of CEInet Statistics Database (https://db.cei.cn/, accessed on 1 May 2022).

Table 1 shows the descriptive statistical analysis of the variables.

## 4. Benchmark Regression Results and Robustness Test

### 4.1. Benchmark Regression Results

Table 2 shows the estimation results of regression Equations (1) and (2) using the two-way fixed effects model. By observing the estimated coefficients of the explanatory variables environmental regulation ER and ER^2^, the study found that: first, the environmental regulation of the Yellow River Basin had a significant effect on the control of air pollutant and CO_2_ emissions, showing a significant inverted “U” relationship; second, the effect of environmental regulations on pollution reduction was significantly better than that of carbon reduction in the Yellow River Basin.

Specifically, Columns (1)–(3) are the pollution reduction effects of environmental regulations. Among them, the first-order term estimation coefficient of environmental regulations on air pollution was positive, while the second-order term estimated coefficient was negative, and both rejected the original hypothesis (with the estimation coefficient being zero) at the significance level of 1%, which indicates that environmental regulation does have an inverted “U”, nonlinear impact on air pollution. The regression results showed that at the initial stage of environmental regulation, every one percentage point increase in environmental regulations corresponded to 11.556 percentage points of SO_2_ emissions (logarithmic); that is, the increase in environmental regulations promoted SO_2_ emissions; when the regulatory intensity crossed a certain inflection point, every one percentage point increase will reduce SO_2_ emissions (logarithmic) by 17.115 percentage points, i.e., it curbs SO_2_ emissions. The reason is that in the short term, the government has imposed a mandatory restriction on the pollution discharge of enterprises, which increases the production cost, and enterprises lack the motivation to invest in pollution control, so that the transformation of industrial green development cannot be achieved. However, in the long run, when the enterprises invest more in pollution control, innovate the production technology, and transform the structures, the worsening situation of air pollution can be effectively curbed.

Columns (4)–(6) show the carbon reduction effect of environmental regulations. It can be seen that the intensity of environmental regulation significantly affected CO_2_ emissions, showing an inverted “U” shape. Specifically, when the intensity of environmental regulation was weak, its estimated coefficient was 1.926, which is significant at the 1% level, indicating that the CO_2_ emissions (logarithmic) will increase by 1.926% for every 1% increase in the intensity of environmental regulation. When the intensity of environmental regulation was high, the estimated coefficient as the core explanatory variable is −2.335, which is significant at the level of 5%, that is, every increase in the intensity of environmental regulation will inhibit CO_2_ emissions (after taking the logarithm) by 2.335 units. In other words, when the intensity of environmental regulation is at a low level, the “green paradox” effect of environmental regulations plays a leading role. In this kind of situation, environmental regulations cannot effectively stimulate enterprise innovation or introduce energy conservation and emission reduction technologies. On the contrary, because of the limitation of the production costs of enterprises, technological innovation investment and energy utilization efficiency are reduced, and carbon emissions continue to grow. When the intensity of environmental regulation crossed the “inflection point” and increased to a higher level, its carbon emission reduction effect gradually became more significant, that is, the “forced emission reduction” effect of environmental regulations starts to be more prominent, gradually offset the negative impact of the “green paradox” effect of environmental regulations, and reduced carbon emissions. At the same time, comparing the estimated coefficients of ER and ER^2^ in column (3) and column (6), it was found that the pollution reduction effect of environmental regulations is significantly better than that of carbon reduction. This may be due to the fact that China’s carbon reduction policy, which focuses on the carbon market, started late, while the implementation of the pollution reduction policy has been carried out for a long time, and the governance effect was more significant because of richer experience.

Furthermore, this paper used the Utest program written by Lind and Mehlum to test the parameter estimation of the “U” shape relationship. The result showed that the relationship between environmental regulations and pollution reduction and carbon reduction in the Yellow River Basin is indeed an inverted “U” shape. In addition, the inflection point of the pollution reduction effect of environmental regulations was 0.338, and the inflection point of carbon reduction effect was 0.412, while the current sample average was 0.105, so the current environmental regulations in most cities of the Yellow River Basin has not played an effective role in environmental governance. Combined with the previous combing, it can be found that, although legislation in the Yellow River Basin has been progressively stricter since 2006 in terms of pollution and carbon reduction, there is perhaps a lack of practical action implementation and weak monitoring and management capacity, so the treatment effect is not up to expectations. The dual dividend of air pollution and CO_2_ emission reduction can only be realized when the environmental regulation intensity crosses the carbon reduction inflection point (0.412), so the situation of pollution and carbon reduction governance in the Yellow River Basin is still severe.

### 4.2. Robustness Test

In order to ensure the robustness of the above baseline results of the environmental regulation effect on pollution reduction and carbon reduction, this paper verified them from four aspects: endogenous test, robustness of estimation methods, robustness of indicators, and other supplementary tests.

#### 4.2.1. Endogenous Test

There may be temporal correlations between air pollutant and CO_2_ emissions. To solve this problem, this paper further used the two-step system GMM regression to test the robustness of the previous conclusions, so as to accurately assess the governance effect of environmental regulations on pollution reduction and carbon reduction. See columns (1)–(2) in Table 3 for specific results. The test statistics showed that the Hansen test values are not significant, indicating that there is no over identification problem, and the tool variables are relatively effective. The *p* value of AR (1) was less than 0.1, and the *p* value of AR (2) was more than 0.1, indicating that the residual terms have first-order autocorrelation but no second-order autocorrelation, and the model setting is reasonable. The regression results showed that the significance of ER and ER^2^ coefficients in the two regression groups was the same as the benchmark results, and the values are close, indicating that the previous conclusions are robust after controlling for the lag of air pollutant and CO_2_ emissions by one period and the endogeneity caused by them.

#### 4.2.2. Changing of the Parameter Estimation Method

The least squares dummy variable (LSDV) estimation and generalized least squares (GLS) estimation are used to regress the benchmark model, as shown in columns (3)–(4) and columns (5)–(6) in Table 3, respectively. It can be found that the size and significance of the ER and ER^2^ estimation coefficients was almost the same as those of the benchmark model, which indicates that the estimation method of the benchmark model is robust.

#### 4.2.3. Replacement Key Metrics

On the one hand, in order to avoid the one-sidedness of the research conclusion due to the selection of measurement indicators, this paper used per capita sulfur dioxide emissions (lnperSO_2_), per capita CO_2_ emissions (lnperCO_2_), weighted averages of PM_2_._5_, and industrial fume and dust emissions (lnpd) as the explained variables to re-estimate the parameters of the benchmark model. According to the results shown in columns (1)–(3) in Table 4, the effect of environmental regulation was still significant. On the other hand, the number of environmental protection practitioners (logarithm) was taken as an alternative indicator of regional environmental regulation to conduct regression analysis again. It can be seen from the results of columns (4) and (5) in Table 4 that the regression coefficients of ER and ER^2^ were still significant, which once again verifies the inverted U-shaped effects of environmental regulations on air pollution and carbon emissions in the Yellow River Basin, and proving that the indicators constructed in this study are reasonable.

#### 4.2.4. Other Robustness Tests

In order to further test the reliability of the above research conclusions, this paper also carried out a series of other robustness analyses. (1) The explanatory variables lagged one period. Considering that the environmental regulations of the previous period may affect the air pollutant and CO_2_ emissions of the current period, the environmental regulations of the previous period were included in the benchmark model as explanatory variables for regression. The estimated results are shown in columns (6) and (7) in Table 4, which verify the robustness of the benchmark regression results. (2) The financial crisis in 2008 slowed down production and reduced energy consumption. In order to eliminate the possible interference of the financial crisis on the conclusions, this paper excluded the sample data of 2008 and 2009. The regression results are as follows: columns (8) and (9) in Table 4, which shows that the environmental regulation of the Yellow River Basin still had a significant inverted “U”-shaped impact on pollution and carbon reduction.

## 5. Transmission Mechanism Identification and Heterogeneity Analysis

### 5.1. Identification of Transmission Mechanism

The above research results showed that environmental regulations have a significant impact on air pollutant and CO_2_ emissions. So, how does it take effect? It has been pointed out that because air pollutants and CO_2_ have the same origin, their emissions are closely related to industrial structure, technological innovation, and energy efficiency [19,21]. Thus, there are three paths for reducing pollution and carbon emissions: Path 1: by rationalizing industrial structure; Path 2: by promoting technological innovation; and Path 3: by improving energy efficiency. Table 5, Table 6 and Table 7 are the test results of the mechanism of environmental regulations on pollution and carbon reduction.

First, Path 1, namely the mechanism of industrial structure (theil) was tested (Table 5). Following the practice of [34], this paper characterized the level of industrial structure transformation and upgrading with the degree of industrial structure rationalization measured by the Thiel index. If the value of the index is 0, it indicates that the industrial structure is in equilibrium; if it is not 0, it indicates that the industrial structure deviates from the equilibrium state and the industrial structure is unreasonable. According to the results of Column (1) and Column (2), the overall relationship between environmental regulations and the Thiel index of industrial structure is in a “U” shape. When it is on the left side of the inflection point, the impact of environmental regulations on the Thiel index is significantly negative at the level of 1%, indicating that environmental regulations can effectively restrain the deviation of industrial structure from equilibrium and promote the rationalization of industrial structure; when it is on the right side of the inflection point, environmental regulations have a negative impact on the rationalization of local industrial structure. A possible explanation is that at the initial stage of environmental regulation by local governments, the entry of high energy-consuming and high polluting industries was restricted by raising barriers for enterprises outside the region [35]. At the same time, under the impact of the policy, some local polluting enterprises responded to environmental protection supervision by reducing production, temporarily shutting down, etc., while some other enterprises actively sought technological innovations to achieve cleaner production by upgrading production technology and reforming product structure [36]. Subsequently, enterprises meeting environmental protection requirements gradually gained more market shares and formed a competitive advantage. Both the reduction of polluting enterprises and the optimization of production structure have promoted the transformation and upgrading of industrial structure. However, with the increasing intensity of environmental regulation and the continuous improvement of pollution discharge standards, the profits of enterprises are reduced due to the increase in pollution control costs, which makes it difficult to invest more funds for the subsequent transformation, prompting the industrial structure to gradually deviate from equilibrium.

Columns (3)–(6) show that the evolution of the rationalization of the industrial structure had always significantly inhibited the emission of air pollutants and CO_2_, and the impact on them is similar as seen from the coefficient, which means that the adjustment of the industrial structure in the Yellow River Basin has formed a long-term mechanism for reducing pollution and carbon emissions.

Secondly, environmental regulation can affect pollution and carbon reduction through Path 2, namely technological innovation (Tec) (Table 6). This paper adopted “(government investment in science and technology/total government financial expenditure) × 0.5 + (total number of patent applications in the year/total population at the end of the year) (The z-score is used to standardize the “total number of patent applications in the year/total population at the end of the year”, i.e., the number of patent applications per capita) × 0.5” to build a technological innovation indicator [36], which more comprehensively reflects that technological innovation is the result of the joint action of innovation R&D input and innovation achievements output. Columns (1)–(4) show that there is a significant lag effect of environmental regulations on technological innovation, and the impact effect is shown as a U-shaped development trend. As technological innovation is limited by the improvement of supporting facilities, the output of innovation achievements, and many other aspects, and these aspects are difficult to achieve immediate results in the short term, so the implementation of environmental regulation policies cannot significantly affect current technological innovation [37]. The impact of environmental regulation policies lagging behind by one period on technological innovation has changed from insignificant to significant, which also shows that technological innovation cannot simply rely on the increase of government R&D investment [38], but also depends on long-term effective policy guidance and guarantee mechanisms. The U-shaped trajectories of different intensities of environmental regulation and technological innovation can be explained from the following aspects: on the left side of the U-shaped track, the negative impact of environmental regulation on local technological innovation comes from two aspects: one is the crowding out effect of innovation investment, and the other is the crowding out effect of investment [39]. When the government’s environmental regulation is weak, the increase in pollution control costs of enterprises has squeezed out their productive investment and R&D investment, making the crowding out effect of environmental regulation on innovation investment greater than the incentive effect. When the environmental regulation is gradually increased to the “inflection point”, the market concentration is increased due to the withdrawal of some high energy consumption enterprises in the early stage, and resources are reallocated among enterprises focusing on innovation. In the face of fierce market competition and diminishing marginal revenue, enterprises will gradually increase the intensity of technology R&D in order to improve output and profits. At this time, the incentive effect of environmental regulation on technological innovation is greater than the crowding out effect.

Therefore, in the long run, strict and reasonable environmental regulations have a certain incentive effect on the technological innovation of enterprises [40]. Columns (5)–(8) show that technological innovation has shown a very stable “technological dividend”, and its pollution and carbon reduction effects were significant at the 1% and 5% levels, respectively, indicating that technological progress in the Yellow River Basin has a positive impact on ecological management and high-quality economic development in the region.

Table 7 shows the test results of Path 3, i.e., energy efficiency (EE) mechanism. To test this mechanism, on the basis of relevant research [41,42], this paper selected human capital, capital stock, and energy consumption as input variables, considered gross regional product as the expected output, and used industrial SO_2_, smoke, and dust and wastewater emissions as unexpected outputs. The SBM directional distance function and Global Malmquist Lounberger (GML) index was used to calculate the energy efficiency of the Yellow River Basin region. As this indicator considers both economic benefits and environmental benefits, it can better reflect the sustainability of the economy. The results of columns (1) and (2) show that the impact of environmental regulation on energy efficiency presents a U-shaped trend, which is consistent with the test results of Path 2. When the regulatory intensity is low, each increase of one unit will cause a decrease of 0.337 in energy efficiency; when the intensity is high, each additional unit will increase energy efficiency by 0.481, and the regression coefficients were significant, indicating that higher intensity environmental regulation was more effective in improving energy efficiency. The reasons are: at the initial stage of regulation, the cost of environmental governance caused by environmental regulations accounted for only a small part of the total cost of enterprises, and enterprises lacked the motivation to carry out green technology innovation to achieve energy conservation and emission reduction [43]. At the same time, the rise in environmental protection costs of enterprises squeezed their production inputs, leading to a decline in economic output and profit margins. In the short term, it is impossible to encourage enterprises to effectively conduct technological R&D, which will hinder the improvement of energy efficiency [44]. With the increase of the intensity of environmental regulation, enterprises face more severe survival pressures: on the one hand, they should reduce pollution emissions; on the other, they should reduce production costs. This situation forces them to pay more attention to energy conservation and emission reduction in the production process, and then force enterprises to improve energy efficiency [45].

The regression results in columns (3) to (6) show that the improvement of energy efficiency has a significant synergistic effect on the emission reduction of local air pollutants and CO_2_. This is because the improvement of energy efficiency can certainly reduce the input and consumption of energy under the condition that the output remains stable, or reduce pollution emissions under the condition that the input remains stable, thus reducing environmental loss in economic development. Therefore, it can be used as a key point for pollution and carbon reduction in the Yellow River Basin.

Based on the above analysis, this paper has identified three action paths of environmental regulation on pollution and carbon reduction. Furthermore, by comparing the estimated coefficients of pollution reduction effect and carbon reduction effect of the three mechanisms in Table 5, Table 6 and Table 7, the second finding in the benchmark regression is supplemented to explain the deep reason why the pollution reduction effect of environmental regulation in the Yellow River Basin was significantly better than that of carbon reduction, which can be attributed to the optimization of industrial structure, the enhancement of technological innovation, and the improvement of energy efficiency.

### 5.2. Heterogeneity Analysis

The above mechanism tests showed that environmental regulations in the Yellow River Basin can reduce pollution and carbon emissions by influencing industrial structure, technological innovation, and energy efficiency. On this basis, this study further explored the heterogeneous impact of environmental regulations on pollution and carbon reduction governance from four aspects in terms of urban characteristics (including the degree of marketization and resource dependence, see Table 8) and urban attributes (whether a city belongs to a national urban agglomeration, see Table 9; whether a city belongs to the upper, middle, or lower reaches, see Table 10).

#### 5.2.1. Differences in Urban Characteristics

First, through the previous research on mechanisms, it was confirmed that environmental regulations can affect pollution and carbon reduction through industrial structure and technological innovation, and the level of industrial restructuring and technological innovation is related to the level of marketization [46]. Then, the effect of environmental regulations may differ depending on the regional marketization level. Based on this, this paper used 2006 as the benchmark and divided the sample into two groups of high and low marketization by the mean value of the marketization index for testing [47]. The results are shown in columns (1) to (4) in Table 8. Compared with column (1) and column (3), there were a significant inverse “U” relationship between environmental regulations and air pollutant emissions in the two groups of samples. In comparison with columns (2) and (4), in the samples with a high degree of marketization, the relationship between environmental regulations and CO_2_ emissions was also in the inverted U-shape, while in the samples with a low degree of marketization, there was no significant relationship. However, by comparing the two columns of ER and ER^2^ estimation coefficients, it was found that the promotion or inhibition effect of environmental regulations on air pollutant emissions is stronger in the samples with a higher degree of marketization.

The reasons for this difference may be: the improvement of marketization has strengthened market competition, forcing enterprises to carry out pollution control and technological innovation under the pressure of survival. The market mechanism also affects the allocation of resources. Under a mature and perfect market mechanism, the requirements of green development guide the flow of factor resources to more environmentally friendly industries [48], so that enterprises meeting the requirements of green production have more market shares. When the market mechanism is not perfect, the resources cannot be effectively allocated, which leads to low utilization efficiency, and ultimately leads to the “resource curse” effect, which makes the environmental regulations less effective.

Moreover, the degree of dependence of the regional resource industry affects the formulation of its environmental regulation policies, which in turn has an indirect impact on pollution and carbon reduction through environmental regulations [35]. Therefore, based on the practice of [49], the proportion of the number of employees in the mining industry and the total number of employees in the secondary industry was taken as the measurement metrics of resource industry dependence, and the sample cities were divided into resource-dependent ones and non-resource-dependent ones for regression analysis according to the average metrics in 2006. According to the results in columns (5) and (8) in Table 8, it can be seen that: the pollution and carbon reduction effects of environmental regulations vary with the degrees of dependence of resource industries, that is, the governance of air pollutant and CO_2_ emissions in resource-dependent regions was significantly effective, while the impact on CO_2_ emissions in non-dependent regions was not significant. Through calculation, the average intensity of environmental regulation in resource-dependent regions and non-dependent regions was 0.106 and 0.104, respectively, and both are on the left of the inflection point (it can be seen from Table 1 that the inflection points for the environmental regulation intensity and lnSO_2_ was 0.338, and that with lnCO_2_ was 0.412) of the inverted “U” curve, but the former is stronger than the latter. It can be seen from this that the requirements of sustainable development have strengthened the environmental regulations in resource-based areas. By strictly limiting enterprises’ emission behavior, strengthening technological R&D, and developing cleaner production, the regional energy consumption structure and energy efficiency have been optimized [50], thus significantly affecting the regional air pollutant and CO_2_ emissions. In addition to government subsidies, preferential bank loans and other policy support, the “resource curse” effect of resource-dependent cities in the Yellow River Basin has been alleviated, and the extensive development model with high energy consumption and high emissions is gradually changing to an intensive one.

#### 5.2.2. Differences in Urban Characteristics

First of all, this paper conducted regression analysis on the sample cities according to whether they belong to a national urban agglomeration (up to now, China has approved 10 state-level urban agglomerations, including the following cities in this article: Zhengzhou, Luoyang, Kaifeng, Nanyang, Anyang, Shangqiu, Xinxiang, Pingdingshan, Xuchang, Jiaozuo, Zhoukou, Xinyang, Zhunadian, Hebi, Puyang, Luohe, Sanmenxia, Changzhi, Jincheng Yuncheng, Liaocheng, Heze, Xian, Baoji, Xianyang, Tongchuan, Weinan, Shangluo, Yuncheng, Linfen, Tianshui, Pingliang, Qingyang, Hohhot, Baotou, Ordos, Yulin, Lanzhou, Xining, Haidong, Baiyin, Haibei, Jinan, Qingdao, Yantai, Zibo, Weifang, Dongying, Weihai, Rizhao, etc.), and the corresponding parameter estimation results are shown in Table 9. There are two findings: first, the cities included in a national urban agglomeration are significantly better than other cities in terms of pollution and carbon reduction; second, by comparing the estimated coefficients of ER and ER^2^ in columns (1) and (2) in Table 9 with the corresponding coefficients in columns (3) and (6) in Table 2, it was found that the coefficients are close, so it is believed that the national urban agglomeration in the Yellow River Basin has driven the pollution reduction and carbon reduction in the whole basin. The reason is that as the core unit of regional economic development, urban agglomeration can not only optimize the allocation of resources in a wider range, so as to control the growth of pollutants while increasing output, and that important policies such as pollution prevention and energy transformation can be implemented; at the same time, it can form a joint force to promote local green development and innovative investment, and has a radiating and driving effect on the surrounding cities [51].

Secondly, this paper further conducted regression analysis on the samples by upstream, midstream, and downstream regions (the divided upstream region includes Qinghai, Ningxia, Inner Mongolia, and individual cities in Gansu; the midstream region includes Shaanxi, Shanxi, Henan, and individual cities in Gansu; and the downstream region includes Shandong). Table 10 shows that the environmental regulations of the Yellow River Basin was significantly unbalanced among regions. From the regression results, this difference was mainly reflected in the governance of CO_2_ emissions in the upper, middle, and lower reaches: Environmental regulations in the middle reaches had a significant impact on CO_2_ emissions, showing an inverted “U” relationship of rising first and then falling, while the upper and lower reaches had no significant impact on carbon emission governance. The reason for this difference may be that the economic base and resource endowment of cities along the Yellow River Basin are different, and the effectiveness of environmental regulations is inherently different. Comparing the three regions, we found the following characteristics: (1) In the upstream region, the population is small, the ecological environment is good, the industrial development is still in the initial stage, and it is a low emission area. (2) The region of the middle reaches has rich energy resources and a relatively mature industrial system. Previous studies have shown that in the upper, middle, and lower reaches of the Yellow River Basin, only the industrial agglomeration in the middle reaches played a positive role in its high-quality economic development. With its economic transformation and development and the promotion of ecological environment protection, pollution and carbon reduction measures were effective. (3) In the lower reach region, the economy is relatively more developed. But some large coal resource and industrial provinces such as Shandong Province have problems such as the industrial structure being biased towards heavy industry, insufficient technological innovation capacity, and slow development of clean energy [52]. Although the pollution control investment in these provinces was higher than other provinces, most of the provinces in the basin could not achieve a positive drive to energy efficiency, and the ecological environment problem could not be effectively improved [53].

## 6. Conclusions and Policy Recommendations

The Yellow River Basin is an important ecological security barrier in China and an important area for production activities and economic development. However, for a long time, the Yellow River has been supporting the development mode of high energy consumption and high pollution emissions in the whole basin with limited resources and fragile ecosystems. Therefore, clarifying the current environmental governance situation in the Yellow River Basin has an important practical significance for promoting the green transformation of the region and thus contributing to high-quality economic development. In this context, this paper used the data of 69 prefecture-level cities in the Yellow River Basin from 2006 to 2018 to explore the effects of pollution and carbon reduction of environmental regulations in the basin using a two-way fixed effects model, and then conducted an empirical study on the mechanisms and heterogeneity, hoping to provide some decision-making guidance for subsequent pollution prevention and control while evaluating the effect of environmental governance.

There were three key findings in the article.

First, the impact of environmental regulations in the Yellow River Basin on air pollution and CO_2_ emissions has an inverted U-shape, and during the investigation period of the samples, the intensity of regulation was still on the left side of the U-shape, and the inflection point of environmental regulations to curb pollution and CO_2_ emissions has not yet appeared. The article proved this conclusion by a number of robustness tests such as the endogenous test, robustness of estimation methods, robustness of indicators, and other supplementary tests.

Second, the mechanism results showed that environmental regulations in the Yellow River Basin can reduce pollution and carbon emissions by influencing industrial structure, technological innovation, and energy efficiency: (1) the relationship between environmental regulation and the Theil index of industrial structure is U-shaped. The current intensity of environmental regulations has promoted the adjustment of the industrial structure, while the latter has a negative inhibitory effect on pollutant and carbon emissions. That is, environmental regulation can achieve pollution and carbon reduction through optimizing the industrial structure. (2) Technological innovation in the Yellow River Basin has shown a very stable “technological dividend”, that is, strict and reasonable environmental regulations have promoted the improvement of the local ecological environment through technological innovation of enterprises. (3) The deepening of environmental regulation intensity will significantly improve energy efficiency and reduce pollution and CO_2_ output with the development of the economy. (4) the optimization of industrial structure, enhancement of technological innovation, and improvement of energy efficiency all have better inhibitory effects on air pollutants than CO_2_ emissions, which is also the deep reason why the pollution reduction effect of environmental regulations in the benchmark regression was significantly higher than the carbon reduction effect. Third, the heterogeneity results showed that the effect of environmental regulations on reducing pollution and carbon emissions was more significant in regions with a high degree of marketization and resource dependence, in regions in a national urban agglomeration, and in regions in the middle reaches of the Yellow River Basin, while environmental regulation in other regions only shows significant effects on reducing pollution, while its effect on carbon reduction still has much room for improvement.

The above conclusions have certain policy implications for promoting pollution and carbon reduction in the Yellow River Basin:

First, strengthen the implementation and supervision of environmental policies. From the previous combing, we can find that the environmental policies in the Yellow River Basin have been improved in recent years, but the environmental management effect is still far from the expected. Therefore, the implementation of environmental policies should be strengthened, and an ecological civilization performance evaluation and accountability system should be established for officials to ensure that the policies are implemented. At the same time, the supervision and review of enterprises should be strengthened to avoid enterprises’ stealthy discharge behavior.

Second, the effectiveness of environmental regulation policies is mainly achieved by optimizing industrial structure, promoting technological innovation and improving energy efficiency. Therefore, the government should continue to implement relevant policies to optimize these three paths. On the one hand, the government should accelerate the elimination of outdated production capacity, increase support for emerging industries and new energy industries, strengthen green infrastructure construction, and promote the gradual rationalization and upgrading of industrial structures. On the other hand, it is necessary to encourage enterprises to strengthen technology innovation and technology transformation with reasonable policies—such as necessary subsidies, etc. In addition, the energy efficiency of the Yellow River Basin is generally low. It is necessary to build a sustainable energy system with new energy and renewable energy as the main supply, gradually eliminating the dependence on coal resources.

Third, the environmental regulation effect in the Yellow River Basin is heterogeneous in different geographical locations and on different levels of resource dependence and capacity utilization. Therefore, in the process of accelerating pollution reduction and carbon reduction, all cities should adjust measures based on local conditions and focus on certain aspects. The downstream region should improve its technology and management level and eliminate enterprises with low production capacity, heavy pollution, and backward production mode; the upstream region can develop deep processing industries and increase investment in R&D; the midstream region should focus on optimizing the industrial structure, curb the blind expansion of high energy consumption and high pollution industries, and encourage enterprises to innovate energy-saving technologies. At the same time, the government will further strengthen support for resource-dependent areas, guide production factors to gather around clean industries, and weaken the dependence of urban development on fossil fuels.

## Figures and Tables

**Table 1:** Variable descriptive statistics.

Variable	Meaning	Sample Size	Mean	Standard Deviation	Maximum	Minimum
lnCO_2_	CO_2_ emissions	897	15.457	1.085	18.327	11.841
lnSO_2_	Emission of air pollutants	897	10.685	1.081	12.728	6.707
ER	Environmental regulation intensity	897	0.105	0.084	0.729	0.002
lnpgdp	Economic development level	897	10.378	0.680	12.165	8.296
lnfd	Financial development level	897	10.034	1.024	12.724	7.720
lnopen	Opening degree	897	13.347	1.806	17.790	7.835
lnpop	resident population at the end of the year	897	5.983	0.656	7.138	4.291
gov	Government R&D investment	897	20.909	3.885	37.000	5.265

**Table 2:** Benchmark regression results.

Variable	Pollution Reduction Effect of Environmental Regulations (lnSO_2_)			Carbon Emission Reduction Effect of Environmental Regulations (lnCO_2_)		
	(1)	(2)	(3)	(4)	(5)	(6)
ER	11.686 ***	11.734 ***	11.556 ***	2.039 ***	2.051 ***	1.926 ***
	(18.63)	(18.69)	(18.50)	(3.36)	(3.39)	(3.19)
ER^2^	−17.479 ***	−17.516 ***	−17.115 ***	−2.663 **	−2.632 **	−2.335 **
	(−15.23)	(−15.26)	(−14.97)	(−2.40)	(−2.38)	(−2.11)
lnpgdp	0.095	0.108	0.006	0.587 ***	0.693 ***	0.629 ***
	(0.78)	(0.83)	(0.05)	(4.98)	(5.52)	(4.87)
lnfd		0.081	0.092		−0.024	−0.011
		(1.15)	(1.31)		(−0.35)	(−0.17)
lnopen		−0.044	−0.056 *		−0.085 ***	−0.094 ***
		(−1.46)	(−1.84)		(−2.89)	(−3.20)
lnpop			1.166 **			1.143 **
			(2.50)			(2.53)
gov			−0.019 ***			−0.012 *
			(−2.91)			(−1.87)
Urban effect	Control	Control	Control	Control	Control	Control
Time effect	Control	Control	Control	Control	Control	Control
_cons	8.786 ***	8.433 ***	2.960	9.203 ***	9.468 ***	3.539
	(6.96)	(6.41)	(0.94)	(7.53)	(7.46)	(1.16)
N	897	897	897	897	897	897
R^2^	0.888	0.888	0.891	0.896	0.897	0.898
Utest		Lower bound	Upper bound	Lower bound	Upper bound	
	Interval	0.002	0.729	0.002	0.729	
	Slope	11.488	−13.398	1.916	−1.479	
	*t*−value	18.516	−11.976	3.191	−1.366	
	Inflection point	0.338		0.412		
	*t*-value = 11.98			*t*-value = 1.37		
	*p* > |*t*| = 0.000			*p* > |*t*| = 0.086		

Note: *, **, and *** are significant at 10%, 5%, and 1% levels, respectively; robust standard errors are shown in brackets.

**Table 3:** Robustness test 1 (Endogeneity test and Replacement estimation method).

Variable	System GMM Estimation		LSDV Estimation		GLS Estimation	
	lnSO_2_	lnCO_2_	lnSO_2_	lnCO_2_	lnSO_2_	lnCO_2_
	(1)	(2)	(3)	(4)	(5)	(6)
L.lnSO_2_	1.037 ***	0.721 ***				
	(0.072)	(0.045)				
ER	9.762 ***	1.989 ***	11.556 ***	1.926 *	13.239 ***	3.833 ***
	(3.648)	(0.678)	(4.99)	(1.83)	(18.34)	(9.43)
ER^2^	−45.145 ***	−2.643 **	−17.115 ***	−2.335 *	−18.619 ***	−4.608 ***
	(14.370)	(1.198)	(−3.61)	(−1.70)	(−10.13)	(−4.25)
lnpgdp	0.768	0.038	0.006	0.629 **	−0.210 ***	0.099 **
	(0.942)	(0.163)	(0.03)	(2.55)	(−3.01)	(2.50)
lnfd	−0.780	0.358 ***	0.092	−0.011	−0.027	0.432 ***
	(0.656)	(0.105)	(0.54)	(−0.07)	(−0.52)	(16.53)
lnopen	0.175 *	−0.112 **	−0.056	−0.094	0.141 ***	0.175 ***
	(0.093)	(0.047)	(−1.19)	(−1.59)	(6.72)	(13.85)
lnpop	−0.942	0.250 ***	1.166	1.143	−0.374 ***	0.238 ***
	(0.628)	(0.070)	(1.36)	(1.44)	(−7.61)	(8.85)
gov	0.035 **	0.030	−0.019 *	−0.012	0.024 ***	−0.005
	(0.015)	(0.020)	(−1.70)	(−1.24)	(3.13)	(−1.42)
_cons	1.635	−0.395	3.174	3.437	11.962 ***	6.146 ***
	(1.525)	(1.179)	(0.51)	(0.63)	(19.88)	(19.23)
Urban effect			Control	Control	Control	Control
Time effect			Control	Control	Control	Control
AR1(p)	0.0329	0.000				
AR2(p)	0.194	0.781				
Hansen(p)	0.140	0.197				
N	828	828	897	897	897	897
R^2^			0.891	0.898		

Note: *, **, and *** are significant at 10%, 5%, and 1% levels, respectively; robust standard errors are shown in brackets.

**Table 4:** Robustness test 2 (Replacement metrics and others).

Variable	Replacement Explained Variable			Replacement Explanatory Variables		Explanatory Variables Lag by One Period		Shortened Sample Period	
	lnperSO_2_	lnperCO_2_	lnpd	lnSO_2_	lnCO_2_	lnSO_2_	lnCO_2_	lnSO_2_	lnCO_2_
	(1)	(2)	(3)	(4)	(5)	(6)	(7)	(8)	(9)
ER	11.556 ***	1.926 ***	6.196 ***	2.593 ***	1.252 **	8.598 ***	2.441 ***	11.839 ***	2.153 ***
	(18.50)	(3.19)	(16.42)	(3.72)	(2.25)	(11.45)	(3.73)	(16.61)	(3.21)
ER^2^	−17.115 ***	−2.335 **	−5.478 ***	−0.148 ***	−0.060 *	−12.324 ***	−3.248 ***	−17.516 ***	−2.736 **
	(−14.97)	(−2.11)	(−7.93)	(−3.70)	(−1.87)	(−9.15)	(−2.77)	(−13.76)	(−2.28)
lnpgdp	0.006	0.629 ***	−0.104	0.032	0.614 ***	−0.090	0.509 ***	−0.023	0.636 ***
	(0.05)	(4.87)	(−1.29)	(0.20)	(4.74)	(−0.55)	(3.56)	(−0.15)	(4.43)
lnfd	0.092	−0.011	0.110 ***	0.008	−0.062	0.051	−0.035	0.069	−0.055
	(1.31)	(−0.17)	(2.61)	(0.10)	(−0.91)	(0.62)	(−0.49)	(0.89)	(−0.75)
lnopen	−0.056 *	−0.094 ***	−0.092 ***	−0.022	−0.076 **	−0.057	−0.099 ***	−0.063 *	−0.078 **
	(−1.84)	(−3.20)	(−4.99)	(−0.60)	(−2.55)	(−1.56)	(−3.08)	(−1.81)	(−2.39)
lnpop	0.166	0.143	0.486 *	1.345 **	0.986 **	0.874	0.730	0.957 *	1.052 **
	(0.36)	(0.32)	(1.73)	(2.39)	(2.19)	(1.57)	(1.51)	(1.81)	(2.11)
gov	−0.019 ***	−0.012 *	0.007 *	−0.024 ***	−0.013 **	−0.016 **	−0.014 **	−0.022 ***	−0.013 *
	(−2.91)	(−1.87)	(1.72)	(−3.08)	(−2.04)	(−2.04)	(−1.96)	(−2.92)	(−1.83)
_cons	2.960	3.539	4.581 **	−8.252 *	−1.237	6.268 *	7.581 **	4.852	4.270
	(0.94)	(1.16)	(2.40)	(−1.67)	(−0.31)	(1.67)	(2.31)	(1.35)	(1.26)
Urban effect	Control	Control	Control	Control	Control	Control	Control	Control	Control
Time effect	Control	Control	Control	Control	Control	Control	Control	Control	Control
N	897	897	897	883	883	828	828	759	759
R^2^	0.920	0.915	0.869	0.843	0.900	0.867	0.897	0.885	0.894

Note: *, **, and *** are significant at 10%, 5%, and 1% levels, respectively; robust standard errors are shown in brackets.

**Table 5:** Mechanism analysis results 1: Industrial structure mechanism.

Variable	Impact of Environmental Regulation on Industrial Structure		Impact of Industrial Structure on Pollution and Carbon Reduction			
			Pollution Reduction Effect		Carbon Reduction Effect	
	Theil	Theil	lnSO_2_	lnSO_2_	lnCO_2_	lnCO_2_
	(1)	(2)	(3)	(4)	(5)	(6)
ER	−0.447 ***	−0.414 ***	11.644 ***	11.498 ***	2.134 ***	2.022 ***
	(−3.48)	(−3.28)	(18.54)	(18.34)	(3.44)	(3.31)
ER^2^	0.714 ***	0.659 ***	−16.197 ***	−15.833 ***	−2.739 **	−2.499 **
	(3.04)	(2.86)	(−14.12)	(−13.82)	(−2.41)	(−2.24)
theil			−0.621 ***	−0.544 ***	−0.667 ***	−0.521 ***
			(−3.62)	(−3.11)	(−3.93)	(−3.06)
Control variables	No control	Control	No control	Control	No control	Control
Urban effect	Control	Control	Control	Control	Control	Control
Time effect	Control	Control	Control	Control	Control	Control
_cons	0.331 ***	2.317 ***	9.930 ***	3.658	15.471 ***	5.578 *
	(32.57)	(3.65)	(132.01)	(1.16)	(208.04)	(1.81)
N	884	884	884	884	884	884
R^2^	0.868	0.875	0.893	0.895	0.897	0.901

Note: *, **, and *** are significant at 10%, 5%, and 1% levels, respectively; robust standard errors are shown in brackets.

**Table 6:** Mechanism analysis results 2: Technological innovation mechanism.

Variable	Impact of Environmental Regulation on Technological Innovation				Impact of Technological Innovation on Pollution and Carbon Reduction			
					Pollution Reduction Effect		Carbon Reduction Effect	
	Tec	Tec	Tec	Tec	lnSO_2_	lnSO_2_	lnCO_2_	lnCO_2_
	(1)	(2)	(3)	(4)	(5)	(6)	(7)	(8)
ER	−1.286 **	−0.866			11.479 ***	11.424 ***	1.969 ***	1.861 ***
	(−2.02)	(−1.45)			(18.52)	(18.45)	(3.20)	(3.08)
ER^2^	1.915	1.074			−17.161 ***	−16.951 ***	−2.498 **	−2.255 **
	1.915	(0.98)			(−15.15)	(−14.97)	(−2.22)	(−2.04)
L.ER			−1.446 **	−1.498 **				
			(−2.24)	(−2.43)				
L.ER^2^			2.031 *	2.228 **				
			(1.75)	(2.02)				
Tec					−0.169 ***	−0.153 ***	−0.102 ***	−0.074 **
					(−4.97)	(−4.21)	(−3.03)	(−2.09)
Control variables	No control	Control	No control	Control	No control	Control	No control	Control
Urban effect	Control	Control	Control	Control	Control	Control	Control	Control
Time effect	Control	Control	Control	Control	Control	Control	Control	Control
_cons	0.701 ***	−7.237 **	0.782 ***	−9.357 ***	9.891 ***	1.854	15.357 ***	3.001
	(14.05)	(−2.39)	(15.18)	(−3.03)	(183.18)	(0.59)	(286.86)	(0.98)
N	897	897	828.000	828	897	897	897	897
R^2^	0.793	0.820	0.820	0.839	0.891	0.893	0.894	0.899

Note: *, **, and *** are significant at 10%, 5%, and 1% levels, respectively; robust standard errors are shown in brackets.

**Table 7:** Mechanism analysis results 3: Energy efficiency mechanism.

Variable	Impact of Environmental Regulation on Energy Efficiency		Impact of Energy Efficiency on Pollution and Carbon Reduction			
			Pollution Reduction Effect		Carbon Reduction Effect	
	EE	EE	lnSO_2_	lnSO_2_	lnCO_2_	lnCO_2_
	(1)	(2)	(3)	(4)	(5)	(6)
ER	−0.335 **	−0.337 **	11.818 ***	11.620 ***	2.179 ***	1.985 ***
	(−2.24)	(−2.26)	(18.82)	(18.56)	(3.57)	(3.31)
ER^2^	0.487 *	0.481 *	−16.490 ***	−16.044 ***	−2.848 **	−2.482 **
	(1.91)	(1.90)	(−14.36)	(−14.01)	(−2.55)	(−2.26)
EE			−0.307 **	−0.307 **	−0.753 ***	−0.749 ***
			(−2.29)	(−2.30)	(−5.77)	(−5.86)
Control variables	No control	Control	No control	Control	No control	Control
Urban effect	Control	Control	Control	Control	Control	Control
Time effect	Control	Control	Control	Control	Control	Control
_cons	1.033 ***	1.076	10.047 ***	2.727	16.041 ***	5.173 *
	(72.35)	(1.64)	(67.26)	(0.87)	(110.34)	(1.72)
N	884	884	884	884	884	884
R^2^	0.206	0.210	0.892	0.895	0.899	0.904

Note: *, **, and *** are significant at 10%, 5%, and 1% levels, respectively; robust standard errors are shown in brackets.

**Table 8:** Results of heterogeneity analysis based on different city characteristics.

Variable	Highly Marketized City		Low Marketized City		Resource Dependent City		Non-Resource-Dependent City	
	lnSO_2_	lnCO_2_	lnSO_2_	lnCO_2_	lnSO_2_	lnCO_2_	lnSO_2_	lnCO_2_
	(1)	(2)	(3)	(4)	(5)	(6)	(7)	(8)
ER	12.593 ***	2.670 ***	11.173 ***	0.507	9.218 ***	2.110 **	18.289 ***	0.971
	(12.11)	(3.46)	(14.18)	(0.57)	(11.14)	(2.34)	(16.70)	(1.05)
ER^2^	−19.903 ***	−3.048 **	−15.420 ***	−0.862	−12.335 ***	−2.875 *	−33.724 ***	0.731
	(−10.37)	(−2.14)	(−10.85)	(−0.54)	(−9.06)	(−1.94)	(−13.94)	(0.36)
Control variables	Control	Control	Control	Control	Control	Control	Control	Control
Urban effect	Control	Control	Control	Control	Control	Control	Control	Control
Time effect	Control	Control	Control	Control	Control	Control	Control	Control
_cons	0.938	17.291 ***	3.502	−9.304 **	6.596 *	−8.428 *	−6.710	16.324 ***
	(0.18)	(4.58)	(0.85)	(−2.02)	(1.67)	(−1.95)	(−1.34)	(3.86)
N	416	416	481	481	442	442	442	442
R^2^	0.862	0.919	0.913	0.896	0.907	0.897	0.898	0.911

Note: *, **, and *** are significant at 10%, 5%, and 1% levels, respectively; robust standard errors are shown in brackets.

**Table 9:** Results of heterogeneity analysis based on different city attributes (whether it belongs to a national urban agglomeration or not).

Variable	National Urban Agglomeration City		Non-National Urban Agglomeration City	
	lnSO_2_	lnCO_2_	lnSO_2_	lnCO_2_
	(1)	(2)	(3)	(4)
ER	11.455 ***	2.282 ***	11.038 ***	0.890
	(14.05)	(2.85)	(10.36)	(0.85)
ER^2^	−16.247 ***	−3.170 **	−17.427 ***	−0.438
	(−10.85)	(−2.16)	(−9.48)	(−0.24)
Control variables	Control	Control	Control	Control
Urban effect	Control	Control	Control	Control
Time effect	Control	Control	Control	Control
_cons	3.012	3.041	−3.199	5.888
	(0.77)	(0.80)	(−0.52)	(0.98)
N	585	585	312	312
R^2^	0.902	0.890	0.855	0.898

Note: **, and *** are significant at 10%, 5%, and 1% levels, respectively; robust standard errors are shown in brackets.

**Table 10:** Results of heterogeneity analysis based on different city attributes (upper, middle, and lower reaches of the Yellow River).

Variable	Cities in the Upper Reach		Cities in the Middle Reach		Cities in the Lower Reach	
	lnSO_2_	lnCO_2_	lnSO_2_	lnCO_2_	lnSO_2_	lnCO_2_
	(1)	(2)	(3)	(4)	(5)	(6)
ER	19.400 ***	2.118	10.610 ***	2.078 ***	17.644 ***	1.113
	(9.68)	(0.74)	(13.88)	(2.79)	(10.00)	(0.87)
ER^2^	−32.603 ***	−4.145	−14.396 ***	−2.730 **	−33.197 ***	1.087
	(−7.51)	(−0.67)	(−11.15)	(−2.17)	(−9.97)	(0.45)
Control variables	Control	Control	Control	Control	Control	Control
Urban effect	Control	Control	Control	Control	Control	Control
Time effect	Control	Control	Control	Control	Control	Control
_cons	−2.653	−23.873 **	11.915 ***	10.828 ***	−23.360 *	19.270 *
	(−0.39)	(−2.45)	(3.16)	(2.95)	(−1.73)	(1.96)
N	130	130	546	546	221	221
R^2^	0.876	0.809	0.915	0.907	0.825	0.878

Note: *, **, and *** are significant at 10%, 5%, and 1% levels, respectively; robust standard errors are shown in brackets.

## Data Availability

The datasets used and analyzed in the current study are available from the corresponding author upon reasonable request.
